# One-Step Synthesis of Silver Nanoparticles Embedded Polyurethane Nano-Fiber/Net Structured Membrane as an Effective Antibacterial Medium

**DOI:** 10.3390/polym11071185

**Published:** 2019-07-15

**Authors:** Bishweshwar Pant, Mira Park, Soo-Jin Park

**Affiliations:** 1 Department of Chemistry, Inha University, 100 Inharo, Incheon 402-751, Korea; 2 Department of Bioenvironmental Chemistry, College of Agriculture & Life Science, Chonbuk National University, Jeonju 54896, Korea

**Keywords:** tannic acid, Ag NPs, polyurethane, electrospinning, antibacterial

## Abstract

A new and straightforward route was proposed to incorporate silver nanoparticles (Ag NPs) into the surface of polyurethane nanofibers (PU NFs). Uniform distribution of in situ formed Ag NPs on the surface of PU NFs was achieved by adding AgNO_3_ and tannic acid in a PU solution prior to the electrospinning process. The synthesized nanofiber mats were characterized with state-of-the-art techniques and antibacterial performances were tested against *Staphylococcus aureus (S. aureus)* and *Escherichia coli (E. coli)* bacteria. The cytocompatibility and cell behavior were studied by using fibroblast cells. Following this preparation route, Ag/PU NFs can be obtained with excellent antibacterial performance, thus making them appropriate for various applications such as water filtration, wound dressings, etc.

## 1. Introduction

Electrospinning has recently gained extensive interest as a simple, inexpensive, and efficient technique for fabricating polymeric fibers (nanometer to micrometer in diameter) by applying high electric fields [1,2]. The electrospun polymeric fibers possess a variety of advantageous properties such as a high surface area-to-volume ratio, high porosity, and interconnected porous structures, which make them attractive for potential application in various fields [2,3]. In recent years, a variety of polymers (synthetic and natural) and their composites with inorganic particles have been electrospun into fiber form for various applications such as filtration, wound dressing, biosensors, drug delivery, tissue engineering, and so forth [3,4,5]. Despite the advantages, the electrospinning process has several limitations to overcome, for example, the optimization of the variables, the slow fiber production rate, the use of organic solvents, high voltage, etc. [6,7]. Furthermore, it is challenging to fabricate electrospun nanofibers with diameters less than 10 nm [7]. Since Ding’s group successfully prepared nano-net like fibers via the electrospinning technique [8], benefits from the relatively small diameter (nano/sub-nano) fibers have evoked a deluge of interest from researchers due to their enhanced surface area/volume ratio and mechanical properties [9]. In the past few years, different research groups have developed polymeric nanofibers with a spider-net like structure via the electrospinning technique to achieve the desired functions [1,9,10,11].

Polyurethane (PU), a thermoplastic polymer, has been intensively spun into nanofibers because of its processability, biocompatibility, and stability [4]. It has been widely applied in biosensors, protective clothing, and biomaterials due to its good mechanical properties and water insolubility [4,12,13]. Recently, the function of PU nanofiber membranes has been widened by incorporating various active components on it. For example, various therapeutic agents such as antibiotics, drugs, polysaccharides, and proteins have been loaded onto these fibers for biomedical applications [4,14,15]. Furthermore, inorganic nanoparticles (NPs) such as silver (Ag), titanium dioxide (TiO_2_), copper (Cu), zinc oxide (ZnO), and fly-ash (FA) have also been incorporated into PU nanofibers to impart new functionalities [10,12,16,17]. Recently, our group prepared salicylic acid loaded PU nanofiber membranes for a wound-dressing application [4].

Ag has been known as an antiseptic since ancient times [18]. Recently, Ag NP embedded polymeric nanofibers have gained significant attention as an antimicrobial agent [3,19,20,21]. In addition, it has also been reported that Ag NPs promote wound healing by decreasing the inflammatory response. Therefore, Ag embedded polymeric nanofiber mats can be considered as a non-toxic and environmentally friendly material in biomedical applications [22]. Generally, two approaches are used to load Ag NPs on electrospun polymer nanofibers [3]: (i) through dispersing previously formed Ag NPs in the polymer solution, and (ii) using post-treatment processes such as UV irradiation, thermal or the chemical reduction of electrospun nanofibers containing AgNO_3_. Since the synthesis process involves multiple steps and requires toxic chemicals, the preparation of a Ag/polymeric composite fiber that is easy and eco-friendly is an important task. Recently, the synthesis of Ag NPs with various natural products including plant extracts has gained extensive interest as a green synthesis [23,24]. In this regard, several groups have prepared Ag NPs through tannic acid (TA) mediated synthesis [25,26,27,28,29,30,31]. TA, a polyphenolic compound that is abundantly found in nearly all plants [32], is a reducing and stabilizing agent that can be used for reducing Ag ions to metallic Ag NPs [25]. In addition, the large number of phenolic hydroxyl groups in TA is also expected to have good antibacterial properties [4,33,34].

In this work, we demonstrate a novel one-step route to prepare a PU nanofiber/net structure membrane with uniformly embedded Ag NPs. A DMF/THF solvent mixture (1:1) containing TA and AgNO_3_ was used to prepare a 10% PU solution and was electrospun to achieve Ag NPs embedded on the nano-net fiber structures of PU. The antibacterial performance of the prepared membrane was studied. Considering the known physical, chemical, and antibacterial properties, it might fulfill the requirements of antimicrobial nanofiber mats for inhibiting microbial growth.

## 2. Experimental

### 2.1. Materials

Polyurethane pellets (Skythane X595A-11, Lubrizol, Seoul, Korea), tetrahydrofuran (THF), and *N*,*N*-Dimethylformamide (DMF) were purchased from Showa Chemicals Ltd., Tokyo, Japan. Tannic acid (TA) was obtained from Sigma-Aldrich, Seoul, Korea. All chemicals were used as received without further purification.

### 2.2. Fabrication of Ag/PU Electrospun Nanofiber Mats

Initially, 75 mg of TA was dissolved in a mixed solvent system of 18 g of THF/DMF (1:1 by weight). The pH of the solution was 8.3. Next, 25 mg of AgNO_3_ was added to the solution and stirred for 1 h at room temperature. To this mixture, 2 g of PU pellets was added to make a 10% PU solution and then stirred for 6 h. The nanofiber mat was prepared by the electrospinning technique. During electrospinning, the applied potential, needle-to-collector distance, and solution feed rate were kept at 18 kV, 15 cm, and 0.5 mL/h, respectively. The electrospinning process was carried out at room temperature. The as-spun nanofiber mat was collected in a polyethylene sheet and vacuum dried at 30 °C for 6 h. For comparison, a pristine PU nanofiber mat (PU NFs), a PU nanofiber mat containing TA (TA/PU NFs), and a AgNO_3_/PU nanofiber mat without the use of TA were also prepared under identical conditions.

### 2.3. Characterization

The morphologies of the as-prepared nanofibers were investigated by field-emission scanning electron microscopy (FESEM, S-7400, Hitachi) and transmission electron microscopy (JEM-2010, JEOL, Tokyo, Japan). For crystallinity determination, a x-ray diffractometer (XRD) was used (Rigaku Co., Tokyo, Japan). Mechanical properties were studied by using a Universal Testing Machine (AG-5000G, Shimadzu, Kyoto, Japan). UV–Vis spectra were recorded by using a UV-visible spectrometer (Lambda900, Perkin-Elmer, Waltham, MA, USA).

### 2.4. Antibacterial Activity Test

The antibacterial performance of the as-synthesized nanofibers (PU NFs, TA/PU NFs, AgNO_3_/PU NFs, and Ag/PU NFs) was investigated by using Gram-positive *Staphylococcus aureus (S. aureus)* ATCC 29231 and Gram-negative *Escherichia coli (E. coli)* ATCC 52922 bacteria by a zone inhibition method in accordance with previous studies with slight modification [35,36]. The pristine PU NFs mat was considered as a control. At the beginning, the bacteria were cultured in a tryptone soy broth at 37 °C for 12 h. A bacterial preculture suspension of 10^8^ CFU/mL was prepared. All samples were cut into a circular disk shape (6 mm diameter) and washed with a phosphate buffered saline (PBS) solution and sterilized by UV light. From the stock bacterial suspension, 100 µL was taken and spread on a tryptone soy agar plate. Then, the samples were gently placed over the plate. After incubating for 24 h at 37 °C, the agar plate was inspected visually and the results recorded. The zone of inhibition was recorded by measuring the diameter of the inhibition area around the disk.

### 2.5. Biocompatibility Test

The cell viability in the presence of pristine PU NFs and Ag/PU NFs was investigated using Donjindo’s cell counting kit-8 in accordance with our previous study [4]. Briefly, after sterilization, the electrospun scaffold samples were transferred to a 48-well plate. The samples were rinsed with phosphate buffer saline (pH = 7), followed by incubation in culture medium overnight under a humidified atmospheric condition at 37 °C. Next, the NIH-3T3 fibroblast cell suspension (10,000 cells/well, DMEM/high glucose supplemented with 10% FBS, and 1% penicillin-streptomycin) was dispensed into the scaffolds and incubated in a 5% CO_2_ atmosphere at 37 °C for the designated time. The medium was changed every two days. The cell proliferation was monitored on the second, third, and fifth days. For this purpose, 30 µL of CCK-8 solution was added to each well and incubated for 3 h. After incubation, 100 µL medium was transferred to a 96-well plate from each well and the absorbance was measured at 450 nm using an iMark^TM^ Microplate reader. The cell proliferation was expressed as a percentage of that of the control cells. The cell attachment was examined by performing chemical fixation. After the fifth day of incubation, the scaffolds were rinsed with phosphate buffer saline and subsequently fixed with 2.5% glutaraldehyde for 1 h. Next, the scaffolds were dehydrated with 25%, 50%, 75%, and 100% ethanol for 10 min each. The samples were dried in a laminar flow for 12 h and the morphology examined by SEM.

## 3. Results and Discussion

**Figure 1:**
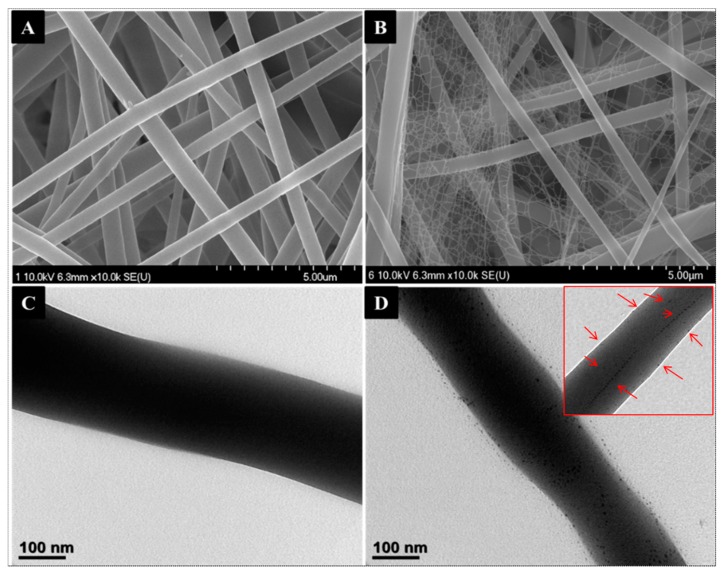
FE-SEM images of pristine PU NFs (**A**) and Ag/PU composite nanofibers (**B**). (**C**,**D**) are their respective Bio-TEM images. The inset in (**D**) shows the Ag NPs embedded in the nanofiber.

The morphologies of the as-synthesized pristine PU NFs and Ag incorporated PU NFs were recorded by FE-SEM and are given in Figure 1. The pristine PU NFs revealed a continuous and smooth fiber morphology without beads (Figure 1A). The average diameter of the PU NF was recorded as 475 ± 50 nm. In the case of the Ag/PU NFs, a clear arrangement of ultrafine nanofibers connected with the main fibers was observed (Figure 1B). The presence of such a spider-web-like nanonet structure along with the main fibers provides a large surface area-to-volume ratio that is beneficial for various applications. The average diameter of the backbone PU NF was found to be 300 ± 50 nm, whereas the spider-web-like ultrafine nanofibers consisted of very fine nanofibers with a diameter range of 10 to 20 nm (Table 1).

**Figure 2:**
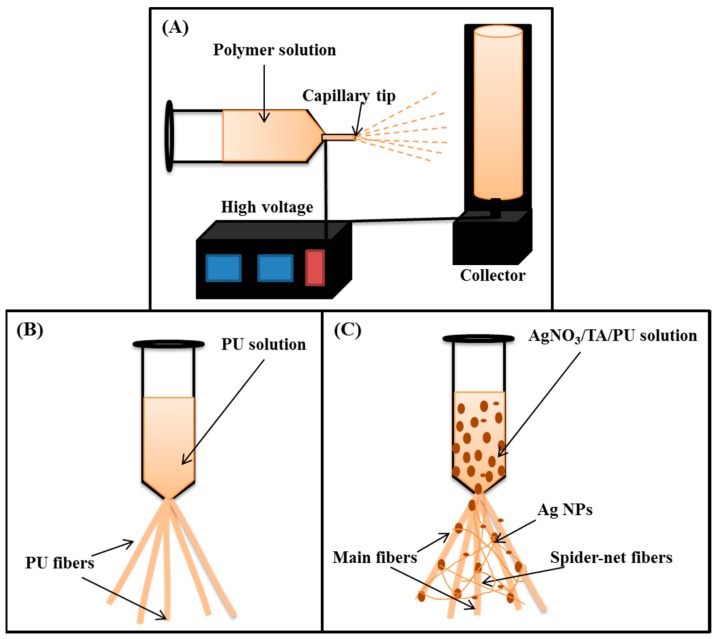
Schematic diagram showing the electrospinning set-up (**A**), mechanism of the formation of PU NFs (**B**), and the spider-net like formation of the Ag/PU NFs (**C**) during electrospinning.

**Figure 3:**
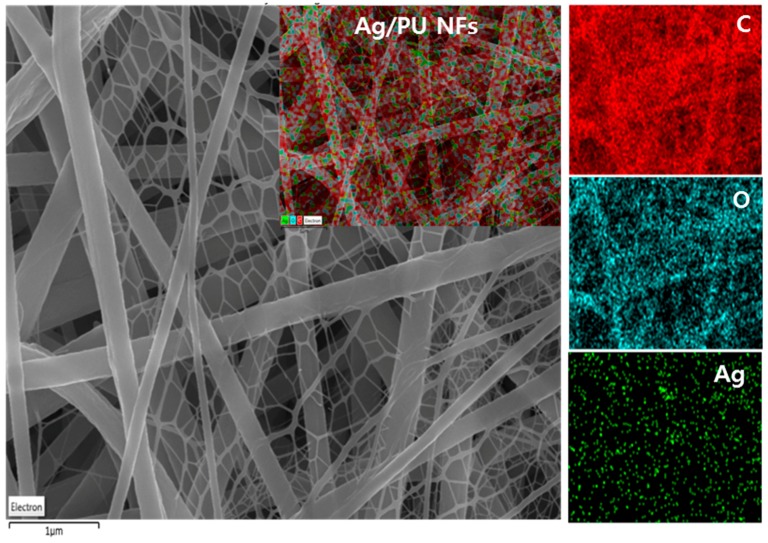
Elemental mapping of the Ag/PU NF sample showing the distribution of Ag NPs along with carbon and oxygen.

Electrospinning is considered as a simple and cost-effective technique for generating continuous nanofibers from a polymeric solution. It involves the application of a high voltage electrostatic field to overcome the surface tension forces of the droplet [37]. A schematic diagram showing the electrospinning set-up is given in Figure 2A. When a high voltage (18 kV) was applied to the PU solution, it induced a charge on the surface of the droplet. The initial hemispherical surface of the solution elongated at the tip of the capillary to form a Taylor cone. Afterward, a jet developed from the cone and travelled toward the rotating collector (Figure 2B). During this process, the solvent evaporated and dry polymer fibers were deposited on the rotating collector. The formation of nano-nets and the deposition of Ag NPs on fibers might be the outcome of the redox reaction that takes place in the solution. Both DMF and TA play a role in reducing the metal precursor. Compared to DMF, TA is an effective reducing and stabilizing agent, therefore, the formation of Ag NPs is mainly attributed to the role of TA. The hydroxyl of the phenolic groups in TA plays an important role in the reduction process. The carboxylic acid groups (COOH) in TA lose a hydrogen atom and form carboxylate ions (COO^−^) during the reduction. Thus formed COO^−^ ions attach to the surface of the Ag NPs to act as a surfactant and stabilize the metal NPs via electrosteric interaction [25,26,38]. It has been reported that alkaline conditions enhance the reducing and stabilizing capacity of the TA, allowing the synthesis of Ag NPs at room temperature [25]. The previously formed Ag NPs in the PU solution increase the electric charge and conductivity of the polymer solution (Table 1), hence it creates more stress on the whipping jet of the metal-rich portion during the phase separation. The high charge density on Ag NPs may force the creation of subsidiary jets, which results in the formation of spider-web-like nano-nets along with the main fibers [10,39] (Figure 2C), whereas no spider-net morphology was observed in the case of pristine PU NFs (Figure 2B). The elemental mapping of the sample showed that the distribution of Ag NPs was all over the nanofibers and also supports the aforementioned mechanism of the formation of the spider-web-like nanofiber (Figure 3). The formation and the attachment of Ag NPs on the PU nanofiber were also confirmed by the TEM images (Figure 1C,D). Compared to the pristine PU NF, the diameter of the fiber was reduced in the Ag/PU NFs, and it can be seen that the Ag NPs were distributed throughout the nanofiber surface. The size of the Ag NPs was 3–6 nm. The Ag NPs embedded into the fiber body can be seen as indicated by the arrows in the inset of Figure 1D.

**Figure 4:**
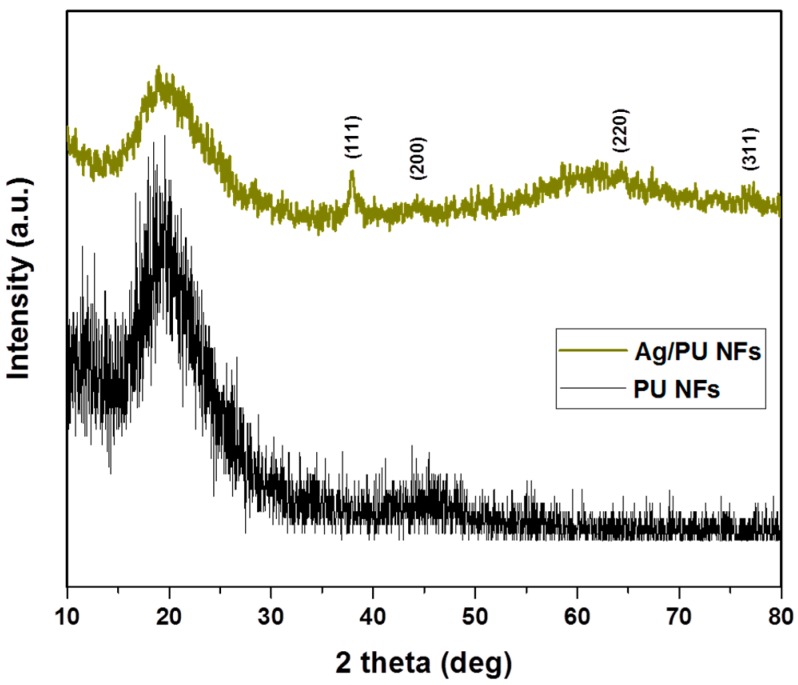
XRD spectra of the Ag/PU NF mat compared to the pristine PU NFs.

**Figure 5:**
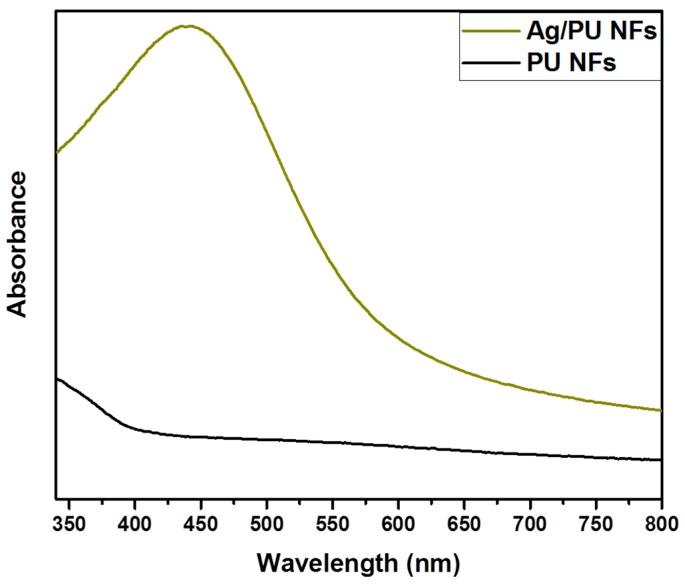
UV–Vis spectra of the Ag/PU NFs and PU NFs.

In order to study the phase and crystalline nature of the composite nanofibers, we carried out an XRD analysis of the Ag/PU NFs and compared it with the pristine PU NFs (Figure 4). A peak at the 2θ values of 20° was observed in both samples, which is the characteristic peak of PU [4,21]. The Ag/PU NF mat showed several extra peaks along with the characteristic peak of PU. In the case of the Ag/PU NFs, diffraction peaks at the 2θ values of 37.3°, 44.27°, 64.42°, and 77.7° were observed, which corresponded to the (111), (200), (220), and (311) crystal planes of Ag, respectively [21,40]. The results obtained from the XRD study confirmed the persistence of crystalline Ag NPs and indicates that TA plays an important role in the reduction of AgNO_3_ and forms pure Ag NPs. UV–Vis spectroscopy was employed to detect the presence of Ag NP in the composite mat (Figure 5). The pristine PU NF mat did not show any absorption peaks in the UV–Vis spectroscopy. On the other hand, the composite mat showed a distinct absorption at about 435 nm due to the plasmonic resonance of the Ag NPs [27,30]. The surface plasmon resonance (SPR) bands in the composite fiber indicate that the Ag (0) had been successfully incorporated into the fiber matrix. The finding from the UV–Vis test simultaneously supports the XRD data.

**Figure 6:**
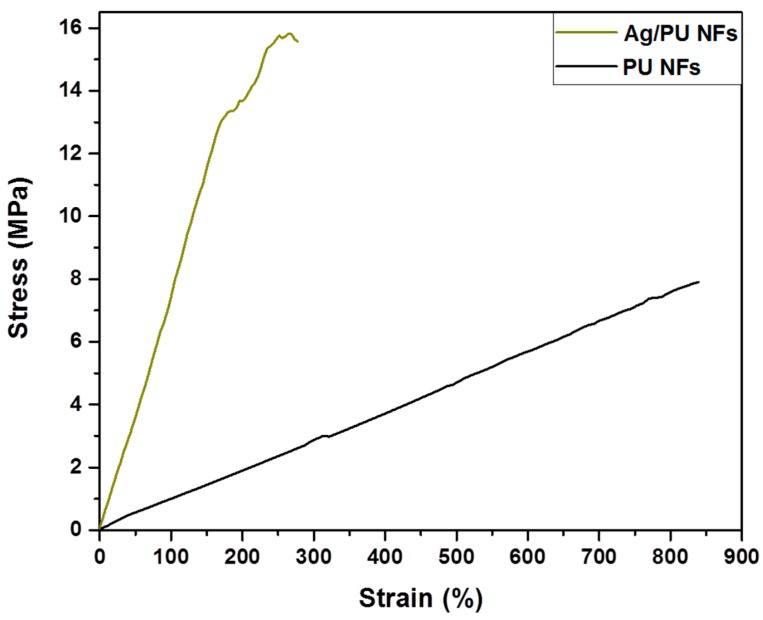
Representative stress–strain curves of the PU and Ag/PU NF mats.

The mechanical strength of nanofibrous mats is considered to be one of the most important characteristics for various applications such as wound dressing, tissue scaffolding, water filtration, etc. [10]. The stress–strain behavior of the as-prepared samples is given in Figure 6, which shows that the Ag/PU composite mat possessed a higher tensile strength compared to the pristine PU NF mat. The enhanced mechanical strength in the case of the composite nanofibers can be mainly attributed its morphology. The interconnected spider-net-like nanofiber network along with the larger backbone fibers led to the enhancement of the mechanical properties [10,41]. In addition, the widespread distribution of Ag NPs into the nanofibers also favors the dissipative mechanism, by which efficient stress-transfer occurs from polymer to inorganic NPs, resulting in improved mechanical properties [42].

**Figure 7:**
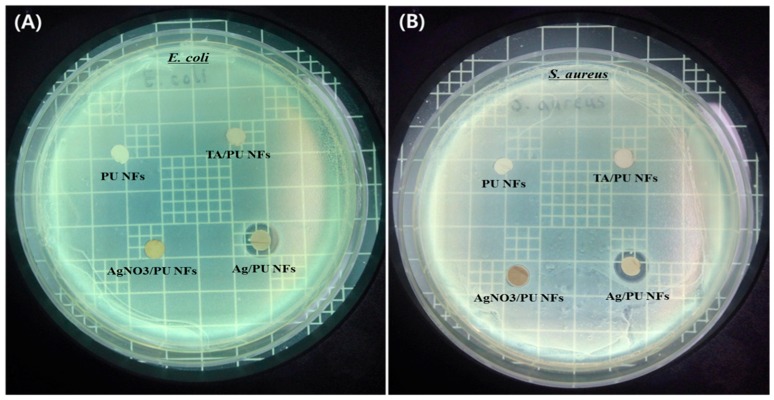
Zone of inhibition test showing the antibacterial performances of different samples against *E. coli* (**A**) and *S. aureus* (**B**).

We evaluated the antibacterial effect by observing the zone of inhibition using *Escherichia coli* *(E. coli)* and *Staphylococcus aureus* (*S. aureus)* bacteria. For the comparison, pristine PU NFs, PU nanofibers with TA, and a PU NF mat with AgNO_3_ (without TA) were also fabricated under identical conditions and their antibacterial performances studied. The zone of inhibition tests for different formulations are given in Figure 7. As shown in Figure 7, the pristine PU NFs, TA/PU NFs, and AgNO_3_/PU NFs did not show any zone of inhibition, suggesting no antibacterial activity. In contrast, the diameter of the inhibition zone was clearly observed for the Ag/PU NFs, which revealed a good antibacterial performance against both bacteria. The diameters of the inhibition zones of the Ag/PU NFs against *E. coli* and *S. aureus* were recorded as 11.4 and 10.8 mm, respectively. In some previous studies, it has been reported that phenolic groups may show antibacterial performance [4,34], however, the TA/PU NFs did not show any antibacterial property, which might be due to the low concentration of TA. Since the precursor solutions of both samples (TA/PU and Ag/PU) contain the same amount of TA and the antibacterial activity was observed only in the case of the Ag/PU NFs, we can conclude that the antibacterial property of the Ag/PU NFs is mainly attributed to the role of Ag NPs in the PU NFs. This indicated that the AgNO_3_ had been significantly reduced to Ag NPs by using TA. It is believed that the Ag NPs can react with DNA, cell membrane, and cellular proteins, leading to the death of the bacterial cell [40]. Ag NPs can anchor to the bacterial cell wall and subsequently penetrate it. This causes structural changes in the cell, leading to death. Furthermore, the formation of free radicals from Ag NPs is also considered as an important factor mechanism by which the cell dies. The Ag ions may interact with the thiol groups of many vital enzymes to inactive them [43]. In addition, the interaction of Ag NPs with DNA also leads to problems in DNA replication, thus terminating the bacteria. It was noticed that the antibacterial effect was slightly higher for *E. coli* than that of *S. aureus*, which was due to the difference in the membrane structures of the two bacteria. Since the cell wall of *E. coli* is thinner than that of *S. aureus*, the Ag NPs had more effect on *E. coli* when compared to *S. aureus* [3]. The obtained results indicated that the as-fabricated Ag/PU NFs were effective for an antibacterial medium.

**Figure 8:**
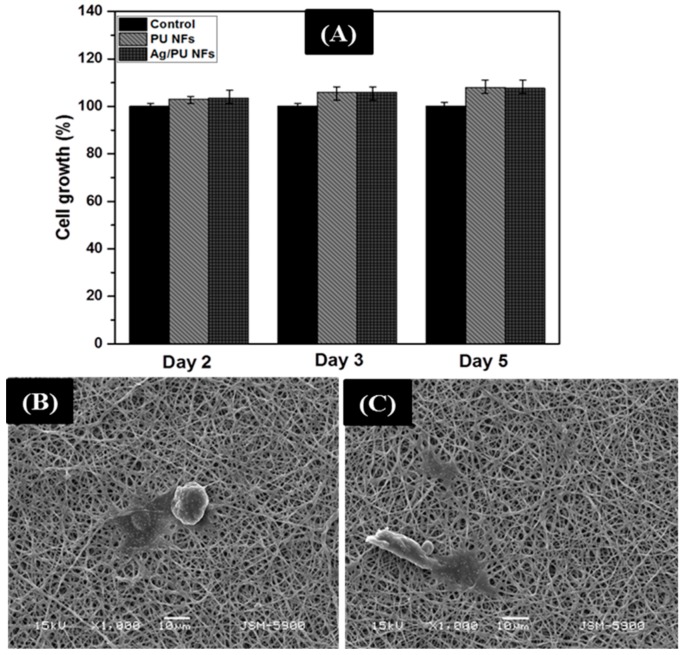
NIH-3T3 cell proliferation on various nanofiber membranes after culture for one, three, and five days (**A**). The viability of the control cells was set to 100% and the viability relative to the control was expressed. (**B**,**C**) represent the SEM images of the cell fixation test on the PU NFs and Ag/PU NFs after five days of incubation, respectively.

The biocompatibility of the nanofibers was studied by using fibroblast cells. Since the Ag/PU NF membrane showed superior antibacterial properties compared to other formulations, we chose the Ag/TU NF mat for the biocompatibility evaluation and compared its performance with the pristine PU NF mat. The results obtained from the MTT assay after two, three, and five days of incubation time are given in Figure 8A. It can be observed that both nanofibers promoted the growth of cells during the entire culture period. Furthermore, to understand the fiber–cell interaction, SEM images were taken after cell fixation and five days of incubation. As shown in Figure 8B,C, the cells were well attached on the surface of the fibers, indicating the proliferation in a healthy manner. From the obtained results, we can conclude that the prepared Ag/PU NF membrane is not toxic and can be applied as an antibacterial and in wound dressing applications.

## 4. Conclusions

We have successfully demonstrated a green strategy to prepare a Ag NP assembled spider-net like PU nanofiber mat as a potential antibacterial medium. The incorporation of Ag NPs onto the composite fibers showed good antibacterial behavior against *S. aureus* and *E. coli* bacteria. The diameters of the nanofibers were found to be in the range of the nano to sub-nanometer scale due to the enhanced conductivity of the polymer solution. More importantly, the Ag NPs were thoroughly distributed not only in the main fiber, but also in the sub-nano fibers. The successful preparation of such a Ag NP loaded spider-web nanofiber PU mat with excellent antibacterial properties and cytocompatibility would provide a reference for designing and developing novel antibacterial materials for various applications such as wound dressing, biofilms, and filtration, etc.

## Figures and Tables

**Table 1:** Conductivity, viscosity, and fiber diameters of the different samples.

Sample	Conductivity (µs/cm)	Viscosity (cP)	Main Fiber Diameter (nm)	Sub-Fiber Diameter (nm)
PU NFs	0.224	705	475 ± 50	-
Ag /PU NFs	5.14	842	300 ± 50	15 ± 6
